# The Misregulation of Cell Adhesion Components during Tumorigenesis: Overview and Commentary

**DOI:** 10.1155/2010/174715

**Published:** 2010-09-30

**Authors:** Claudia D. Andl

**Affiliations:** Department of Surgery and Cancer Biology, Vanderbilt University, Nashville, TN 37232, USA

## Abstract

Cell adhesion complexes facilitate attachment between cells or the binding of cells to the extracellular matrix. The regulation of cell adhesion is an important step in embryonic development and contributes to tissue homeostasis allowing processes such as differentiation and cell migration. Many mechanisms of cancer progression are reminiscent of embryonic development, for example, epithelial-mesenchymal transition, and involve the disruption of cell adhesion and expression changes in components of cell adhesion structures. Tight junctions, adherens junctions, desmosomes, and focal adhesion besides their roles in cell-cell or cell-matrix interaction also possess cell signaling function. Perturbations of such signaling pathways can lead to cancer. This article gives an overview of the common structures of cell adhesion and summarizes the impact of their loss on cancer development and progression with articles highlighted from the present issue.

## 1. Tight Junctions

**Figure 1:**
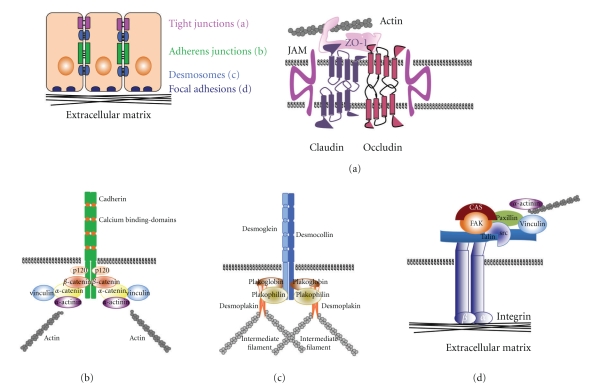
Schematic diagram of tight junctions (a), adherens junctions (b), desmosomes (c) and focal adhesions (d). This is an overview of the interactions of the major components of cell adhesion complexes.

Tight junctions are regulators of the epithelial microenvironment as they are responsible for the formation of paracellular barriers (see Figure 1), [1, 2]. Claudin-based tight junctions and their functions have been analyzed in numerous knockout mouse studies. The loss of claudin-1 or -5 is embryonically lethal due to loss of the barrier function of the skin and loss of the blood-brain barrier [3, 4]. In cancer, claudins can be found to be up- or downregulated depending on the cancer type. Claudin-1 and -7 are downregulated in esophageal cancer [5], but upregulated in others [6, 7]. While the mislocalization of claudin-7 in esophageal squamous cell carcinoma leads to the loss of E-cadherin expression, N-glycosylation of E-cadherin has been shown to stabilize tight junctions [8]. An in-depth review of claudins and cancer can be found in this special issue of the Journal of Oncology (Singh et al., [9]). 

## 2. Desmosomes

Desmosomes are adhesion complexes tethered to the intermediate filament, (see figure 1), [10]. Desmosomal cadherins, the desmogleins, establish the contact to the neighboring cells [11]. Plakoglobin is homologous to *β*-catenin and binds to the same region of the cadherin tail [12, 13]. While Plakoglobin is highly enriched in desmosomes, it can also be localized to adherens junctions in cells that do not have desmosomes, such as endothelial cells [14, 15]. There is evidence that plakoglobin can participate in Wnt signaling as the transcription factor T-cell factor/lymphoid-enhancer factor, TCF-4, contains binding sites for *β*-catenin and plakoglobin [16], and that binding of plakoglobin could hinder transcriptional activity. However, Plakoglobin has been shown to have TCF/LEF-dependent transcriptional activity in *β*-catenin-deficient cell lines [17].

Desmoplakin connects desmosomes through binding of plakoglobin to the intermediate filament. It is downregulated in oropharyngeal cancer [18] and a target of EGF and progesterone in breast cancer [19]. Interestingly, aside from its obvious function in cell adhesion, desmoplakin has been described to regulate microvascular tube formation [20]. Therefore, desmoplakin may be a novel target for the inhibition of tumor angiogenesis.

The desmosomal cadherins, desmoglein 1, and 3, are targets in two autoimmune diseases, Pemphigus foliaceus and Pemphigus vulgaris, respectively [21]. Binding of autoantibodies to desmoglein induces cell dissociation and inhibition of RhoA in a p38 MAPK-dependent pathway causing the hallmark blistering [22]. The implications of desmosomal component loss have been shown in mouse models targeting desmoglein 2 [23] and desmoglein 3 [24, 25], plakoglobin [26] and desmoplakin [27]. Desmoglein 2 knockout mice proved to be embryonically lethal, despite unaffected E-cadherin and *β*-catenin expression [23]. Mice with loss of desmoglein 3 presented the same blistering phenotype as Pemphigus patients [24]. Interestingly, targeted loss of desmoplakin in the epidermis allowed the formation of desmosome-like structures, but epithelial sheet formation was impaired in the face of mechanical stress [27]. 

Effects of desmosomal perturbations on tumorigenesis rarely share the lime light with the well-known consequences of adherens junction loss. However, loss of desmoglein 1 has been associated with poor prognosis in head-and-neck cancer patients [28]. Contrary, Desmoglein 2 upregulation is associated with malignant skin carcinoma including basal cell carcinoma and SCCs in a tissue-microarray-based study [29].

Plakophilins, which are armadillo family members like plakoglobin and *β*-catenin, are structural components of the desmosomal plaque and regulate the strength and integrity of cell contacts by facilitating the interaction with the intermediate filament [30]. Decreased expression of plakophilin 1 promotes cell invasion due to desmosome instability [31]. Furthermore, the inverse correlation of plakophilin expression with tumor grade in head-and-neck SCCs has been documented [32]. Similarly, RNAi (small interference RNA) suppression of plakophilin 3 results in transformation of epithelial cells and accelerated tumor formation as well as lung metastasis in mouse tumor xenografts [33]. 

In addition, other junction types have been identified that use desmosomal components without being desmosomes. A recent review by Pieperhoff et al. [34, 35] highlights composite junctions that connect cardiomyocytes, plakophilin-2-positive junctions in sarcomas as well as the expression of Desmoglein 2 in melanoma. These data together with molecules discussed in the last paragraph of this paper demonstrate that we may not have discovered all types of cell adhesion yet.

## 3. Adherens Junctions

Early on, experiments targeting E-cadherin and *β*-catenin have shown that adherens junction components are essential for normal development. E-cadherin- and *β*-catenin-null embryos display lethality due to primary defects in morphogenetic events such as trophectoderm development and ectoderm formation [36, 37]. Deletion of N-cadherin, VE-cadherin or plakoglobin also leads to embryonic lethality, however at later stages of development [26, 38, 39]. As for *α*-catenin, loss of this gene results in death shortly after birth [40]. Interestingly, lack of E-cadherin in thyroid development or adult tissues can be overcome by upregulation of other cadherins as a mechanism of compensation [41, 42]. In cancer, however, loss of E-cadherin is associated with tumor progression, even if other cell adhesion complexes remain intact. This has been attributed not only to the detrimental effects E-cadherin loss has on the tissue integrity and dissemination of cells “on the loose”, but also to the signaling pathways activated in the absence of E-cadherin [43–45]. Alterations of the cadherin-catenin cell adhesion system and how they relate to cancer have been focus of multiple symposia and meetings resulting in numerous review articles [46] to this date, and where already discussed at the Princess Takamatsu Symposium in 1994 [47–49].

Cadherins interact through their intracellular domain with cytoplasmic proteins, the catenins (see Figure 1), [50, 51]. *β*-catenin mediates the anchoring of adherens junctions to *α*-catenin and other actin-binding proteins, and thereby to the cytoskeleton [52]. The relative amount of cadherin-bound *β*-catenin and free *β*-catenin can tip the balance to induce Wnt signaling [44, 53]. This occurs if free *β*-catenin is not degraded by the ubiquitin-proteasome pathway, but translocated to the nucleus instead to regulate target gene expression in conjunction with members of the T-cell factor/lymphoid-enhancer factor (TCF/LEF) family of transcription factors [54]. Activation of cells with Wntmolecules can inhibit *β*-catenin degradation and allows its accumulation in the cytosol and translocation to the nucleus leading to the activation of genes such as cyclin D1, c-myc, CD44, and others [55, 56]. Constitutive active Wnt signaling either through mutations of *β*-catenin or loss of adenomatous polyposis coli (APC) function frequently leads to cancer [57], as particularly well understood for colon cancer [58].

p120ctn binds cadherins at the juxtamembrane domain of the cytoplasmic tail and prevents their internalization and degradation [51, 58]. Similarly to *β*-catenin, unbound p120ctn can translocate to the nucleus where it binds Kaiso, a zinc finger transcription factor that acts as a transcriptional repressor and tumor suppressor. Once bound to Kaiso, p120ctn relieves the repressor activity of Kaiso by dissociating it from its sequence-specific binding sites [59]. Wnt signaling stabilizes p120ctn and results in Kaiso withdrawal from the nucleus [60–62]. p120ctn also functions as a regulator of cell motility by modulating the activity of Rho GTPases [63] and has been shown to coordinate Rho inhibition through Rac [64]. In this context, a p120ctn isoform has been shown to fail to inhibit RhoA and to promote invasion [65]. In another model, overexpression of P-cadherin has been linked to the activation of the RhoGTPases, Rac1, and Cdc42, through accumulation of p120ctn in the cytoplasm during cell invasion [66]. Furthermore, overexpression of the p120ctn isoform 3A demonstrated cytoplasmic accumulation. This isoform is also associated with cyclin E- and cyclin-dependent kinase 2-colocalization at the site of centrosomes during mitosis [67]. Ablation of p120ctn in the skin also results in mitotic defects and, additionally, a chronic inflammatory response [68]. Conditional knockout in the small intestine and colon disrupts normal barrier function and epithelial homeostasis resulting in phenotypic and morphological changes associated with inflammatory bowel disease [69].

## 4. Cadherins and Cancer Cell Signaling

Cadherins can signal in different ways: they can bind to growth factor receptors and modulate their internalization and downstream pathways. They also activate signaling mediators, such as phosphatidylinositol 3-kinase (PI3K) or small GTPases. Alternatively, they can recruit transcriptional cofactors, such as *β*-catenin or p120ctn, at the cell membrane and thereby negatively control their nuclear translocation.

A number of cadherins has been implicated in cell signaling via interaction with receptor tyrosine kinases: both E-cadherin and N-cadherin interact with FGFR-1. To prevent constitutive or prolonged signaling by FGFR-1, it is sequestered by E-cadherin and internalized [70]. Contrary, complex formation of N-cadherin with FGFR-1 prevents internalization and circumvents degradation. This is known to be one of the mechanisms by which N-cadherin contributes to tumor cell invasion. The switch from E-cadherin to N-cadherin expression occurs during normal developmental processes and is recapitulated in cancer [71, 72].

E-cadherin can also interact with epidermal growth factor receptor (EGFR) [73]. EGFR overexpression is a frequent event in epithelial cancers. EGFR promotes cell motility by phosphorylation of *β*-catenin and plakoglobin leading to the disruption of cell adhesion [74]. At the same time, E-cadherin-mediated inhibition of EGFR activity is an important aspect in tumorigenesis. Somatic mutations of E-cadherin have been linked to increased EGFR activation resulting in activation of Ras [75, 76]. Other studies have found that E-cadherin can cluster EGFR at the cell membrane thereby inhibiting EGFR-mediated signaling [77, 78]. Similarly, desmoglein 1 can suppress EGFR signaling resulting in epidermal differentiation [79].

VE-cadherin is an endothelial specific transmembrane protein concentrated at adherens junctions. Similar to E-cadherin it engages in homophilic cell-cell adhesion. A link to the cytoskeleton is established through the same intercellular partners, *β*-catenin, p120 and plakoglobin [80]. Upon VEGF stimulation, VE-cadherin binds to VEGFR-2 preventing vascular endothelial growth factor 2, VEGFR-2, phosphorylation. This clustering of VE-cadherin with VEGFR-2 blocks cell proliferation by inhibition of MAPK activation [81]. Furthermore, VE-cadherin is required for TGF*β* receptor-mediated TGF*β* signaling. This has been demonstrated through knockdown of VE-cadherin [82], but also as *β*-catenin null-endothelial cells are unable to respond to TGF*β* stimulation [83].

Another interesting aspect is that tumor-inducing viruses alter cell adhesion. In the case of Kaposi-sarcoma-associated herpesvirus, VE-cadherin is targeted inducing endothelial permeability and contributing to the progression and malignancy of this disease [84]. While Kaposi sarcoma-associated herpesvirus induces VE-cadherin degradation, hepatitis B virus HBx-protein disrupts adhesion junctions in asrc-dependent manner [85]. Epstein Barr Virus “attacks” cell adhesion complexes through another mechanism: virus-induced gene silencing [86]. E7 protein of Human Papillomavirus 16 (HPV), for example, augments DNA methyltransferase I activity associated with the silencing of E-cadherin gene expression [87]. Simultaneously, N-cadherin expression is increased [88]. Augmented cell invasion in HPV-infected cells can be suppressed through restoration of E-cadherin and subsequent downregulation of EGFR [89] or ErbB2 [90]. The mechanism on how Src/ABL regulates cell differentiation and invasion in E6/E7-positive cervical cancer is described in this issue by Yasmeen et al., [91]. Another virus-associated protein, Epstein Barr Virus-latent membrane protein 1, also affects the cadherin switch [92].

## 5. Epithelial Mesenchymal Transition (EMT)

Similar to the cadherin switch, epithelial-mesenchymal transition is an important process of development, but is “hijacked” as a mechanism of malignant transformation resulting in mesenchymal-like high motility cells. The spotlight on EMT is warranted by the many signaling pathways (peptide growth factors, Src, Ras, Ets, integrins, Wnt/*β*-catenin, and Notch) involved in the regulation of this process. However, a central node is the downregulation of E-cadherin [93, 94]. Activation of PI3K/Akt is another feature of EMT [95]. Despite its role as a tumor suppressor, TGF*β*1 signaling is often increased in tumor cells and induces EMT, thereby leading to tumor cell invasion [96]. This morphological transition is characterized by extensive changes in the expression of cell adhesion molecules and by a switch from a cytokeratin-rich cytoskeleton to one comprising a mesenchymal cell phenotype, for example, the expression of vimentin and S-100 [97]. The ability of epithelial or carcinoma cells to undergo EMT in culture correlates with cell changes that facilitate invasion and metastasis *in vivo * [98–100]. Increased cell motility and scattering are caused by a downregulation of E-cadherin, mainly through the TGF*β*-induced upregulation of transcriptional repressors such as ZEB1, ZEB2, and Snail. This is accompanied by the decreased expression of ZO-1 and keratins [97, 101, 102].

Focal adhesion kinase (FAK) can also mediate TGF*β*-induced EMT [103]. The induction of mesenchymal migration through FAK signaling and its importance in glioblastoma is discussed by Zhong et al. [104] in this issue of the Journal of Oncology. As EMT results in increased cell invasion, it is accompanied by the digestion of the extracellular matrix and changes in matrix metalloproteinase (MMP) expression. Overall, E-cadherin has been shown to induce the suppression of MMP expression. When restored in motile prostate cancer cells, E-cadherin not only reverted EMT and induced an epithelial phenotype, but also reduced MMP-2 expression levels resulting in decreased cell invasion [96, 105]. Lynch et al. [106] describe in this issue how cleavage of E-cadherin by MMP-7 promotes cell proliferation through activation of RhoA.

The microenvironment is a prominent modulator of tumorigenesis and some of these aspects are covered in this special issue: the modeling of microenvironments *in vitro* (by Ngalim et al. [107]), the tumor-stromal interactions in prostate cancer (by Josson et al. [108]) and the upregulation of laminin-322 by lysophosphatic acid and its effects on colony dispersal (by Yamashita et al. [109]).

More recently, with the advent of microRNAs, small non-coding RNAs (miRNAs) regulating gene expression, an additional level ofprotein translation regulation has been added. A number of miRNAs that inhibit tumor suppressor genes have been identified as well as miRNAs, which negatively affect the translation of oncogenes. Cadherin scan be targets of miRNAs directly or indirectly through the modulation of transcriptional repressors that target cadherins. Ma et al., identified E-cadherin as a direct target of miR-9 [110] leading to activation of *β*-catenin and increased invasion as well as increased tumor angiogenesis via VEGF upregulation. miR-145 is an example of a tumor suppressor miRNA, which silences MUC-1, thereby reducing *β*-catenin and oncogenic cadherin-11 [111]. The miR-200 family gained a lot of attention as it participates in a signaling network with the E-cadherin repressors, ZEB1 and 2 and TGF*β*1, therefore placing it at the center for the regulation of the epithelial phenotype. Another central regulator of cell invasion and metastasis that is upregulated in the absence of E-cadherinis Twist [43]. Twist, as well as ZEB1 and other transcription factors, is thought to induce EMT by suppression of E-cadherin. The data by Onder et al. [43], however, suggest Twist to be downstream of E-cadherin and sufficient to mediate cell invasion and metastasis as well as to prevent anoikis. The authors demonstrated that, while the loss of cell-cell contacts can induce changes in gene expression leading to increased cell invasion, the induction of EMT and its associated gene expression changes only occurs if *β*-catenin is released from the E-cadherin cytoplasmic tail.

## 6. Focal Adhesions

FAK, focal adhesion kinase, is a crucial mediator of integrin and growth factor signaling. FAK resides within focal adhesion complexes, large integrin clusters that mediate crosstalk between the extracellular matrix and the cytoskeleton, where it regulates outside-in signaling (see Figure 1). High levels of FAK in a variety of human cancers have been reported [112, 113], including a study in head and neck squamous cell carcinoma (HNSCC) that shows enhanced FAK signaling at the onset and progression of HNSCC [114]. The increased expression of FAK has been linked to cancer cell migration, proliferation, and survival [115, 116]. Motility defects in FAK-null ES cells [117] can be restored with wild-type FAK, but not with a mutant of FAK lacking the Tyr^397^-phosphorylation site, which is responsible for Src recruitment [118]. Actin rearrangements are responsible for the formation of adhesion complexes that stabilize the leading edge. Leading edge formation and membrane ruffles are regulated by Rho GTPases such as Rac 1 and RhoA [119]. This issue of Journal of Oncology also features paper focusing on the effects of hyperphosphorylated FAK on its localization to focal adhesions (see Hamadi et al. [120]). Two FAK-binding scaffold proteins that mediate Rac1 activity are CAS (p130cas) and paxillin. Paxillin regulates the localization of FAK [121] and possibly regulates Rac1. Interestingly, in the study by Yano et al. [121] the suppression of FAK and paxillin resulted in increased cell migration, presenting FAK as a negative regulator of cell motility in contrast to other reports. Furthermore, the impaired FAK/paxillin signaling cascade prevented N-cadherin-based cell-cell contacts. While E-cadherin has been described to stimulate Rac1 activity [122], N-cadherin is thought to suppress Rac1 activation [123]. The important role of FAK in cancer is supported by the intersection of the FAK and p53 signaling pathways. Not only has the FAK-promoter p53 binding sites, there is also a high correlation between FAK upregulation and p53 mutations [124]. These data demonstrate the regulation of FAK by p53. 

## 7. Summary

While the main components of cell adhesion structures are well defined, recently new players such as the nectins have been identified [125]. Similar to cadherins, nectins bind the cytoplasmic protein afadin and are linked to the actin cytoskeleton [126]. However, nectins can participate in cell adhesion through interaction with cadherins in adherens junctions, ZO-1 or claudins in tight junctions as well as independently [127–129]. Additionally, nectins have been shown to regulate E-cadherin endocytosis [130–132] and to function in migration and polarization [133]. Others include abLIM3, a novel component of adherens junctions [134], and protocadherins, which have multiple functions including neuronal specificity [135, 136] and are therefore not discussed in this issue. Differences in the function and tissue-specific expression patterns of all the cell adhesion molecule family members involved in the pathogenesis of cell transformation make therapeutics challenging. However, knowledge of the crosstalk between signaling pathways and common themes such as the interaction of cell adhesion molecules with growth factor receptors allow new scientific advances. Taken together, new mechanisms of the regulation of cell adhesion structures and their signaling function demonstrate the importance of understanding cell adhesion and its impact on disease (see Cell Junctions, edited by LaFlamme [137]) and tumorigenesis [138].
